# An extended approach to appraise electricity distribution and carbon footprint of bitcoin in a smart city

**DOI:** 10.3389/fdata.2023.1082113

**Published:** 2023-02-02

**Authors:** Ayushi Sharma, Pratham Sharma, Harsh Bamotra, Vibha Gaur

**Affiliations:** Department of Computer Science, Acharya Narendra Dev College, University of Delhi, New Delhi, India

**Keywords:** cryptocurrency, crypto-mining, carbon emission, environment, electricity consumption

## Abstract

A nation cannot sustain a highly productive and efficient population without smart cities. Due to their significant reliance on digital technologies, these cities require a high level of cybercrime protection. Cryptocurrencies have gained significant attention due to their secure and reliable infrastructure. The decentralised cryptocurrency operates in a trust-less environment known as the blockchain, where each network participant has a ledger copy of all transactions. Blockchain technology employs a proven consensus mechanism without requiring establishment of a central authority. But the consensus mechanism requires miner to solve a cryptographic problem by generating random hashes until one of them matches the desired one. This procedure is energy-intensive, and when thousands of miners repeat it to verify a single transaction, a substantial amount of electricity is consumed. Moreover, electricity produces a significant amount of carbon footprint. Patch methodology utilises the data of all hashes created per year and the efficiency of mining hardware over a 10-year period to calculate the Bitcoins energy consumption. Due to a large number of unknown and uncertain factors involved, it is difficult to precisely calculate a single value for electricity consumption and carbon footprint as reported by Patch methodology. The proposed method extends the Patch methodology by adding a maximum and minimum limit to the hardware efficiency as well as the sources of power generation, which can help refine estimates of electricity consumption and carbon emissions for a more accurate picture. Using the proposed methodology, it was estimated that Bitcoin consumed between 38.495 and 120.72 terawatt hours of electricity in 2021 and released between 2.12 and 45.37 million metric tonnes of carbon dioxide. To address the issue of excessive energy consumption and carbon emissions, a significant number of individual miners and mining pools are relocating to energy-intensive regions, such as aluminium mining sites that rely on hydroelectricity for energy generation.

## 1. Introduction

Smart cities are a vision of a megalopolis that can house and manage a highly productive population in a challenging and changing environment. Infrastructure problems in Tier 1 cities like Delhi, Bangalore, Mumbai, and other metros during heavy rains show how important and necessary it is for a city to be well-planned. In these cities, a secure, smart, and digital currency like Bitcoin with its high-end protocol is necessary for the transfer of funds.

Bitcoin has been around for over a decade now, and ever since the genesis block was mined in 2009 by Satoshi Nakamoto (Panda et al., 2021), it has paved the way for several other alternative coins of its kind. It is based on blockchain technology, which is a peer-to-peer network that forms a decentralized ledger that verifies transactions without the need for a central authority. Bitcoin mining employs the proof-of-work consensus mechanism, with the longest ledger in a tie, and promises a secure cyber network that can't be tampered with in any way. This makes it a strong way to handle large-scale transactions. The fact that it doesn't have a central system also shows that it can handle bigger transactions 24 hours a day, seven days a week. Also, because it is open, it is future-proof because there is a community of developers who can fix bugs and open discussion panels to make the technology better as needs and policies change. It has changed the way the economy and finance are practiced on a global scale. But every revolution brings with it several repercussions. In the case of Bitcoin, the major issue that makes it controversial is its carbon footprint. A transaction system needs to have a verification system that stops fraud. Usually, in a fiat economy, these verifications are done by banks, or, to be more specific, a hierarchy of banks, where on top is the central bank, which is affiliated with the central government. But because Bitcoin is decentralized, no one in charge can check for fraud. This means that a verification system must be 100% accurate. Bitcoin achieves this by maintaining a ledger with a verification system. This system is maintained by an entity called “miners.” Miners are independent players who sign up to check new transactions and get paid for them.

For security, Bitcoin uses the SHA-256 algorithm (Gilbert and Handschuh, 2004) to encrypt the transaction. This algorithm converts the transaction data into a “hash code” of 64 digits in length that is unique for every transaction. The main benefit of using the Bitcoin system is that neither a person nor a computer can reverse the hash code. Every transaction provides an encrypted hash code, popularly referred to as “hashes”, and miners compete to decode the hash; whoever decodes it first gets rewarded. Since a hash code cannot be reversed, its decoding is done using a hit-and-trial method in which more than a tera-hash is created per second on a random basis in the network. In the process of mining bitcoin, most of the electricity is used to create hashes at random. Since the majority of the electricity produced all around the globe, even in developed countries like the USA, uses coal and other highly carbon-emitting sources, an electricity-intensive system like Bitcoin cannot help but create a chunk of a carbon footprint. So, the carbon footprint is directly related to electricity consumption and its source of production. The location of the miner makes it easy to figure out the carbon footprint of each mining transaction. However, because cryptocurrency is decentralized and trustless, it is very hard to track down a miner. Many researchers have made multiple assumptions, like the one in the Zumo Methodology (Johnson and Pingali, 2021), which makes the vague assumption that 60% of a miner's revenue goes toward two electricity costs. With a best-case and worst-case scenario, the proposed method makes a more accurate estimate and comes to a more realistic conclusion.

In 2021, each tera hash of Bitcoin mining will use about 50 joules of energy. The proposed study will figure out how much electricity the Bitcoin mining network has used and how much carbon it has released over the past 10 years. This energy crisis in Bitcoin puts its sustainability under the scrutiny of environmentalists and general people alike.

The organization of the paper is as follows: Section 2 presents the related work reported in the literature. In Section 3, the research method is explained, and in Section 4, the results of using the proposed research method are given. Finally, Section 5 concludes the paper.

## 2. Background

People see cryptocurrency as the future of the digital economy, and its market capitalization will reach more than $2 trillion in 2022. It is thought to be the payment method for the next generation of smart cities because it is widely used and well-known. Blockchain technology can be used in smart cities for many things, like insurance, supply chain management, environmental management, keeping track of medical records, managing identities, and making financial transactions (Gade and Aithal, 2020).

In smart cities, Bitcoin is often seen as the currency of the future. Because of this, it is important to evaluate its long-term viability. Hakak et al. (2020) came up with a three-layer plan for smart cities based on the blockchain. The authors said that blockchain technology can be used to protect smart cities because it is open and not controlled by one person or group.

Researchers are paying a lot of attention to the steady rise in energy use and its harmful effects. The Bitcoin network is consuming a huge amount of electricity year after year, leaving behind the power consumption of some countries such as Ireland and Austria (O'Dwyer and Malone, 2014; de Vries, 2018). Marc Johnson and Sahithi Pingali (Gallersdörfer et al., 2021) explain how to measure the electricity usage and carbon emissions for different stakeholders in crypto-mining using the Patch and Zumo methodologies. de Vries (2018) has estimated the power consumption of Bitcoin to range from 2.55 GW to 7.67 GW. The author said that miners will keep making hashes until their marginal costs and marginal products are the same. Gallersdörfer et al. (2021) used a mix of transaction-based and investment-based methods to figure out how much power was used. This method uses the weighted ratio of block and transaction rewards. Miners use powerful and efficient hardware that uses a lot of electricity.

Küfeoglu and Özkuran (2019) examined the performance of 269 different types of hardware, including FPGAs, CPUs, GPUs, and ASICs. According to their study, the historical peak for Bitcoin mining was between 1.3 and 14.8 GW in the two-weeks starting on December 18, 2017. They also calculated power consumption for the year 2018, which ranges between 15.47 and 50.24 TWh. Different hardware models were compared depending on their power consumption and efficiency. Pathirana et al. (2019) concluded that the Bitmain Antminer S9 is the most efficient piece of hardware compared to ASIC and FPGA, while Nvidia GPUs are more expensive and less efficient than ATI GPUs. These pieces of hardware, in turn, contribute significantly to carbon emissions. Mining uses a lot of carbon-rich fuels to make electricity, which is bad for the environment. Bisht et al. (2022) use a machine learning model to find a link between the different sources of production and the amount of dangerous carbon that is released. The power consumption and the carbon footprints for an open-source cryptocurrency, Monero, were computed (Li et al., 2019). They used the network hash rate and the hardware efficiency of the mining machines for the same. Al Kawasmi et al. (2014) presented a model for a decentralized carbon emission for Bitcoin. Similarly Stoll et al. (2019) used mining hardware, facilities, and pools for the purpose. They used the IP addresses of pools, nodes, and devices to figure out the region's carbon footprint. They reported that the carbon emissions range from 23.6 to 28.8 MtCO_2_, which is between what Jordan and Sri Mongolia produce. Kononova and Dek (2020) developed a method for figuring out the carbon footprint of Bitcoin mining based on miners' locations, and the value of carbon emissions were estimated as 44.12 MtCO_2_/year. But a range can be a better way to estimate these parameters than a single number. This research suggests a long-term way to figure out how much energy is used and how much carbon is released.

## 3. Materials and methods

In smart cities, it is very important to have a secured network of blockchain for crypto-mining because block chain is decentralized and keeps detailed records of all transactions. For green computing, it is important to make sure that the electricity needed to run the blockchain network comes from sources that produce less carbon. But previous research has shown that a lot of the energy used in mining comes from carbon-heavy sources like coal and fossil fuels, which release a lot of carbon into the atmosphere (World Nuclear Association, 2021). Because of this, it is important to look at the carbon emissions that come from the mining process and figure out how to cut them down. In the literature, the process for estimating carbon emissions is based on several assumptions that may lead to inconsistent results. An extended framework is proposed to fix this problem and make it clear how much electricity is used and how much carbon is released.

**Figure 1:**
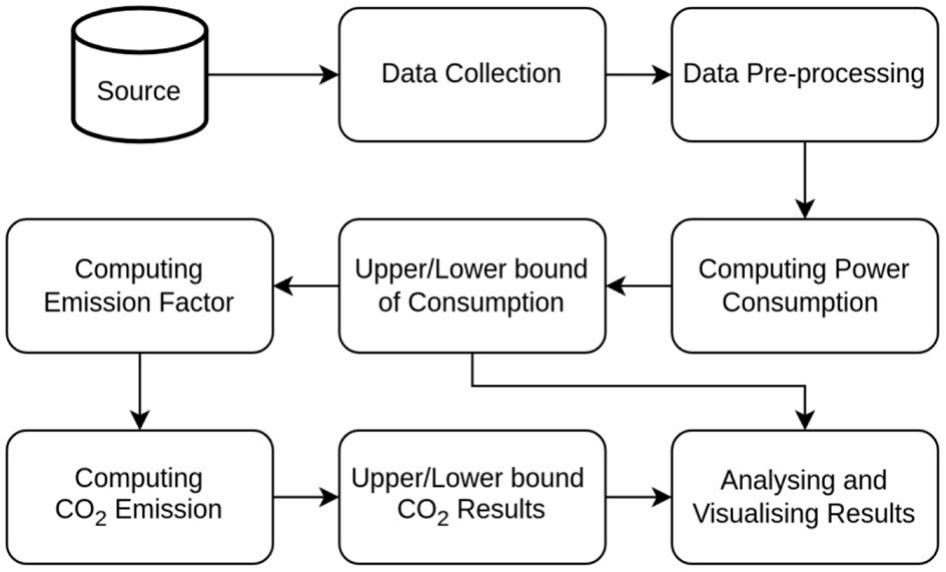
Proposed framework for computing power consumption and carbon emission.

In this section, the proposed framework for figuring out the amount of electricity used to mine cryptocurrencies and their carbon footprints is described. The framework entails collecting and preprocessing the data, followed by determining the ranges for electricity consumption and carbon dioxide emissions during cryptomining. Figure 1 shows the proposed framework, and the steps are briefly explained below:

### 3.1. Data collection

**Figure 2:**
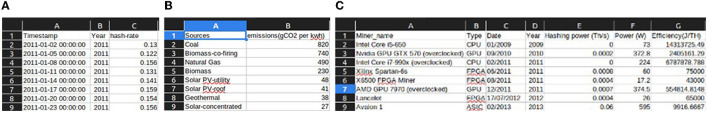
**(A)** Screen-shot of hash-rate dataset. **(B)** Screen-shot of carbon emission dataset. **(C)** Screen-shot of efficiency of hardware dataset.

Data from sources like https://www.blockchain.com/ and https://www.kaggle.com/ was utilized to perform deep analysis. The datasets include hash rate, characteristics of different hardware used for mining and their efficiencies from 2011 to 2021, and the carbon footprints generated per gram of CO_2_/kWh of the various sources. The computational power on the blockchain network is measured by hash rate. To get the best results, the quality of the data is very important. Because of this, the data pre-processing is done with the most relevant and reliable datasets. The hash rate dataset includes the attributes *timestamp, year, and hash rate*. The timestamp shows the record's date and time, the year shows what year the record is from, and the hash rate shows how much computing power the blockchain network had at the given date and time. The names of the sources and the amount of carbon they release (g CO_2_ per KWh) are in the carbon emission dataset. The hardware dataset includes miner_name, type, date, year, hashing power (Th/sec), power (W), and efficiency (J/TH). Miner_name specifies the name of the mining hardware, and type specifies the type of mining hardware, such as CPU, GPU, and so on. The date and year indicate when the hardware was used for mining, and the efficiency indicates the computational power (tera hashes per second) of the mining hardware. Figures 2A–C present screenshots of the aforementioned datasets.

### 3.2. Data pre-processing

The raw data was put into a format that was easy to understand to improve its quality and make it easier to use. Data pre-processing includes cleaning, transformation, filtering, and grouping of data. When data from different sources is put together, there may be duplicates or missing values. To fix this, the Pandas library in Python was used to fill in missing values and remove duplicate or unwanted attributes. Undesired attributes were present in the hash rate dataset, from which only useful attributes were selected, i.e., year and hash rate. Similarly, year and efficiency (J/Th) were selected from the efficiency dataset. As the hash rate and efficiency data were spread over a vast timeline, they were filtered onto a particular timeline for a decade, i.e., 2011 to 2021, and grouped year-wise to improve the accuracy of the data.

### 3.3. Computing power consumption

Numerous theories have been put forth to calculate the electricity consumption for bitcoin mining. The literature reports that the Patch methodology (Li et al., 2019) facilitates a single value for electricity consumption and, hence, has few limitations. So, this work gives a more detailed way to figure out how much electricity Bitcoin mining uses.

The Patch Methodology uses commercially available mining equipment and its efficiency to figure out how much electricity is used every day (Johnson and Pingali, 2021). Miners use power-intensive equipment to verify the crypto transactions on the network; the power consumed by the equipment depends on its efficiency and hash rate. Li et al. (2019) computed electricity consumption as the product of hardware efficiency and the network hash rate of cryptocurrencies. Using the Patch method, the amount of electricity used in 2021 is estimated to be 60 TWh. However, these results are not consistent with the existing literature (de Vries, 2018). In this work, to get a more realistic estimate of how much electricity is used, the limitations of the Patch method are overcome with an extended approach.

As the geographical locations of crypto miners are not known, it may be hard to find a single value for the amount of electricity used for Bitcoin mining. So, this work extends the Patch methodology by adding an upper and lower limit to the amount of electricity used. It also narrows the range of possible solutions.

### 3.4. Estimating upper and lower bounds of power consumption

The lower bound describes what would happen if miners used the most efficient hardware, while the upper bound looks at what would happen if they used the least efficient but most profitable hardware. The upper limit is found by taking the average of the hardware efficiency of the least efficient but most profitable hardware and the network hash rate.

**Figure 3:**
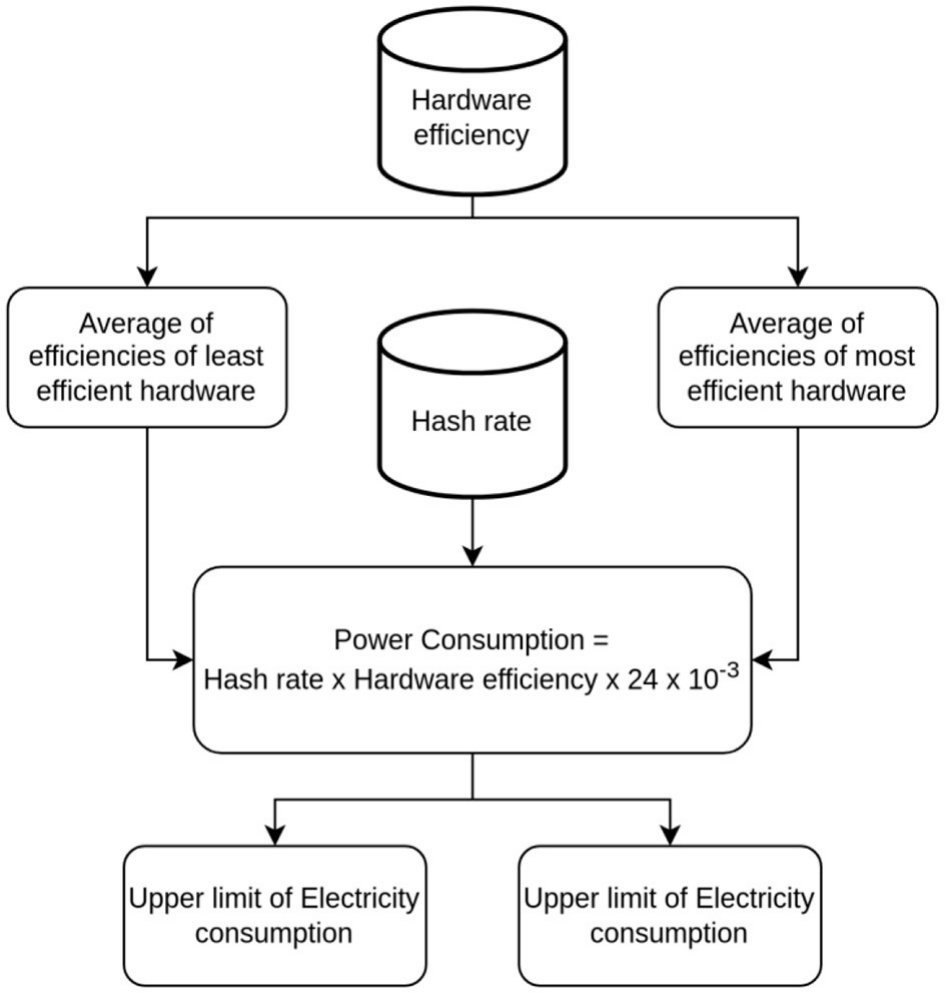
Overview of the proposed approach for computing ranges of electricity consumption.

In the same way, the lower bound is worked out by multiplying the network hash rate by the average hardware efficiency of the most efficient hardware. Figure 3 shows an overview of the proposed method for determining ranges of electricity consumption. The value of electricity consumption is computed as follows:

Electricity Consumption (B) = Hardware Efficiency x Network Hash-rate

$$\begin{array}{l} B_{u}=Ef_{l}\;\times \;Hr\\ B_{l}=Ef_{m}\;\times \;Hr \end{array}$$

where,

Hr = Network hash-rate
B_u_ = Upper Bound, B_l_ = Lower Bound
Ef_l_ = Average efficiency of least efficient hardware
Ef_m_=Average efficiency of most efficient hardware.

$$\begin{array}{l} B÷Nh=Ef\;÷\;\left(3.6\;\times \;106\right)\\ B=\left(Ef\;\times \;Nh\right)÷\left(3.6\;\times \;106\right)\\ B=\left(Ef\;\times \;Hr\;\times \;24\;\times \;3600\right)÷\left(3.6\;\times \;106\right)\\ B=Ef\;\times \;Hr\;\times \;24\;\times \;10^{-3} \end{array}$$

where,

Nh = Number of hashes = Hr x 24 x 3600

After the proposed methods have been applied to determine the results, the results are viewed using Jupyter notebook. The method for calculating the carbon emission is described in the subsection that follows.

### 3.5. Computing the emission factor

To compute the lower and upper bounds of carbon emission, emission factors are used. These factors facilitate the estimation of emissions caused by various sources. For the upper limit of the emission factor, the average number of grams of carbon dioxide per kilowatt-hour (gCO_2_/kWh) from all carbon-intensive sources is used. Similarly, for the lower bound, only the sources with minimal carbon emissions are considered.

### 3.6. Computing carbon emission

When figuring out carbon footprints, the amount of electricity used to mine Bitcoin is taken into account. The calculations are done by multiplying the emission factor by the amount of electricity needed to mine bitcoin, which was calculated above.

Carbon footprints = Emission factor x Electricity consumption

### 3.7. Estimating the upper and lower bound of carbon emission

To get a realistic estimate of the carbon footprint of bitcoin, an upper bound and a lower bound are used to set the emission range. The scenario in which the miners mine from a place that generates electricity from carbon-intensive sources is taken as the upper bound. The lower bound describes a situation in which the miners work from a place where electricity is made with energy sources that leave the least amount of carbon behind, like solar energy, wind energy, or hydro energy. To calculate the lower and upper bounds of carbon emission, emission factors are used. These factors facilitate the estimation of emissions caused by various sources. For the upper limit of the emission factor, the average of gCO_2_/kWh produced by different carbon-intensive sources is used. Similarly, for the lower bound, only the sources with minimal carbon emissions are considered.


$$\begin{array}{l} B_{u}=Ef_{l}\;\times \;Pc\\ B_{l}=Ef_{m}\;\times \;Pc \end{array}$$

where,

Pc = Electricity Consumption
B_U_ = Upper Bound, B_L_ = Lower Bound
Ef_L_ = Average emission of Carbon intensive sources
Ef_M_ = Average emission of green power sources.

### 3.8. Result analysis and visualization

Python's Jupyter notebook and related libraries, like Numpy and Pandas, are used to look at the data and make it easier to understand. Matplotlib and Excel are then used to turn the results of the analysis into a graphical representation of the data. The total electricity consumption from 2009 to 2021 varies between 115 TWh and 331 TWh, while the carbon emission ranges from 5.950 Mt CO_2_ to 127.372 Mt CO_2_.

The proposed framework was used to figure out how much electricity Bitcoin mining uses and how much carbon it releases. The results are presented in the next section.

## 4. Results and discussion

As discussed in the previous section, there is not enough information to estimate the exact number of emissions that bitcoin causes. Many large mining pools conceal information such as hardware demographics and power source distribution to prevent competition. An approximate range of electricity consumption and carbon emissions can be computed to get an idea of the sustainability of the cryptocurrency. The data collected from different sources, like https://www.bitcoin.com/ and https://www.kaggle.com/, was preprocessed. The above-mentioned proposed framework was used to find out how much electricity Bitcoin mining used and how much carbon dioxide it released from 2009 to 2021.

### 4.1. Electricity consumption

**Figure 4:**
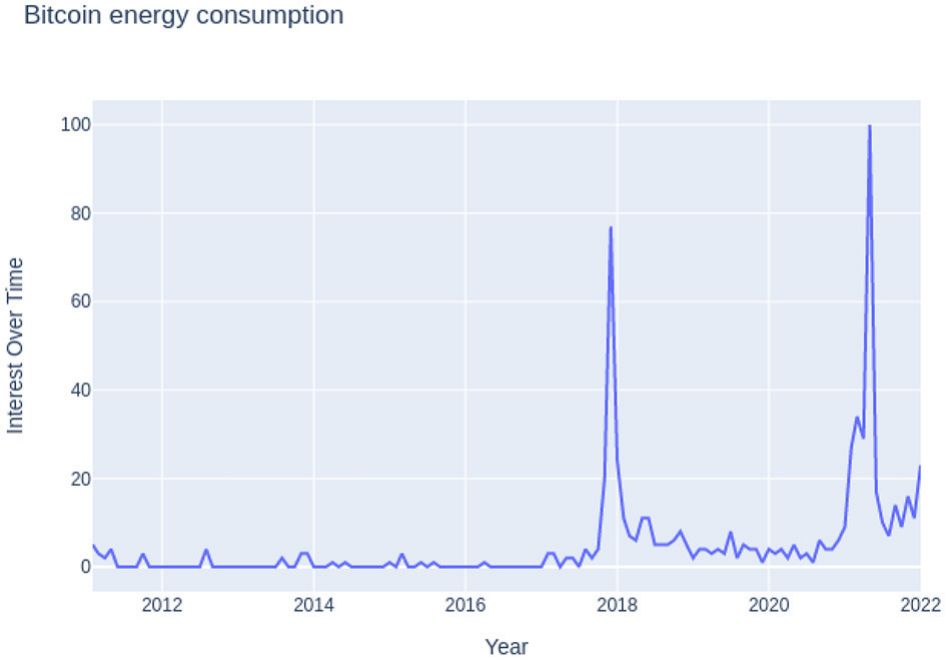
Google searches for “Bitcoin energy consumption” from 2011 to 2021 (Google Trends, 2022a).

Using estimates of the upper and lower limits, the proposed framework was used to figure out how much electricity Bitcoin transactions use per year. Since there isn't enough information about the hardware each miner uses for each transaction, the efficiency of mining hardware can be roughly put into two groups: the least energy-intensive and the most energy-intensive per tera hash for a given year, as presented in Table 1. The table contains the minimum and maximum efficiencies of hardware (J/TH) grouped by year. The total annual hash rate of bitcoin from 2011 to 2021 is given in Table 2. Figure 4 shows the increase in Google searches for “Bitcoin energy consumption.” The x-axis represents the period from 2011 to 2022, and the y-axis represents the number of searches.

**Table 1:** Total annual hash rate of bitcoin from the year 2011 to 2021.

**Year**	**Hardware efficiency (J/TH)**	

	***Min***	***Max***
2011	43000	6787878.788
2012	65000	65000
2013	2000	9916.6667
2014	510.8225	765.6904
2015	273.3615	273.3615
2016	98	258.1481
2017	97.1264	157.5342
2018	45	256.75
2019	39.5	97.037
2020	29.5455	54
2021	30.5	95.6522

**Table 2:** Total annual hash rate of bitcoin from the year 2011 to 2021.

**Year**	**Annual hash-rate**
2011	775.03
2012	1,919.33
2013	173,397.35
2014	17,148,718.45
2015	49,451,630.72
2016	187,022,841.41
2017	763,080,841.38
2018	4,441,195,589.90
2019	8,176,909,956.35
2020	14,623,412,642.11
2021	17,530,042,159.98

A comparative analysis of annual electricity consumption was carried out, and the results of electricity consumption are presented in Table 3. The table specifies the minimum, maximum, and mean values of electricity consumption for the years 2011–21. It can be observed from Table 3 that there was a growth of more than 100% in the carbon emissions of bitcoin mining in 2018, making it the most pivotal year in terms of environmental concerns. Figure 4 shows that more people are searching for “Bitcoin Energy Consumption” on Google, which is also a sign of the trend. This work has taken into consideration the period of 2011–2021 instead of the whole lifecycle of Bitcoin because, in the initial years of Bitcoin, the miners used their devices and household power supplies. These devices might use a lot of electricity per tera hash, but because there are not as many Bitcoin transactions, they might not make a big difference in Bitcoin's carbon footprint.

**Table 3:** Electricity consumption of mining bitcoin (Twh).

**Year**	**Electricity consumption (Twh)**		

	***Min***	***Max***	***Mean of Min max***
2011	0.002399486	0.378777220	0.190588353
2012	0.008982465	0.008982465	0.008982465
2013	0.024969219	0.123805710	0.074387464
2014	0.630716489	0.945403854	0.788060172
2015	0.973308381	0.973308381	0.973308381
2016	1.319633169	3.476130564	2.397881867
2017	5.336301242	8.655215752	6.995758497
2018	14.389473711	82.099941675	48.244707693
2019	23.255131916	57.129322423	40.192227170
2020	31.108034752	56.855828353	43.981931552
2021	38.495972583	120.728671106	79.612321845
Total	115.545247667	331.375711757	223.460479712

### 4.2. Carbon emissions

The decentralized nature of Bitcoin brings many pros; it makes it extremely challenging to calculate the carbon emissions and electricity consumption in maintaining the whole ledger, so estimates are made to put it into a realistic range of values. The proposed method looks at the amount of electricity used in both the worst-case and best-case scenarios. In the best case, it is assumed that the miner is using energy sources that put out little carbon. In contrast, the worst-case scenario is based on the idea that the miner is using power sources that release a lot of carbon. The average carbon emissions for the two types of power sources are shown in Table 4. The table shows the lower and upper limits of the emission factor used for carbon-intensive and green power sources. It can be seen that there is a significant difference between the emissions from both sources. Depending on the region and the technology, the emissions for the same amount of electricity can be different. As a result, average emissions are considered rather than the highest and lowest emissions. Finally, electricity consumption was used for evaluating carbon emissions.

**Table 4:** Average emissions of lower and upper bound energy sources in grams per kilo-watt-hour (Gilbert and Handschuh, 2004).

**Category**	**Emissions (gCO_2_/kwh)**

	***Average of emissions***
Lower Bound	26.625
Upper Bound	570

Table 5 shows the minimum, maximum, and mean carbon emissions of bitcoin mining in millions of metric tons from 2011 to 2021. From the table, we can figure out that the rise in popularity of Bitcoin during the “panic trading period” of lockdown (Béjaoui et al., 2021) led to a 50% increase in the carbon footprint in the year 2021. The panic trading period not only brought the crypto market more into the mainstream economy but also opened the gates for debates about its sustainability over time. This is also confirmed by the increase in Google searches for “Bitcoin Carbon Footprint” as shown in **Figure 7**.

**Table 5:** Minimum, maximum and mean carbon emissions of bitcoin mining in million metric ton from 2011 to 2021.

**Year**	**Carbon emissions**		

	***Min***	***Max***	***Mean of min max***
2011	0.005074	0.108635	0.056855
2012	0.000239	0.005120	0.002680
2013	0.001981	0.042401	0.022191
2014	0.0210	0.4492	0.2351
2015	0.0259	0.5548	0.2904
2016	0.0638	1.3668	0.7153
2017	0.1863	3.9876	2.0869
2018	1.285	27.499	14.392
2019	1.070	22.910	11.990
2020	1.171	25.070	13.120
2021	2.120	45.379	23.749
Total	5.950	127.372	66.66

Environmentalists are concerned about the huge carbon emissions caused by Bitcoin mining. Many other crypto-currencies are already using low-emission processes such as proof-of-stake to handle the issue. Since no one owns Bitcoin, it is extremely difficult to make changes to the consensus mechanism.

**Figure 5:**
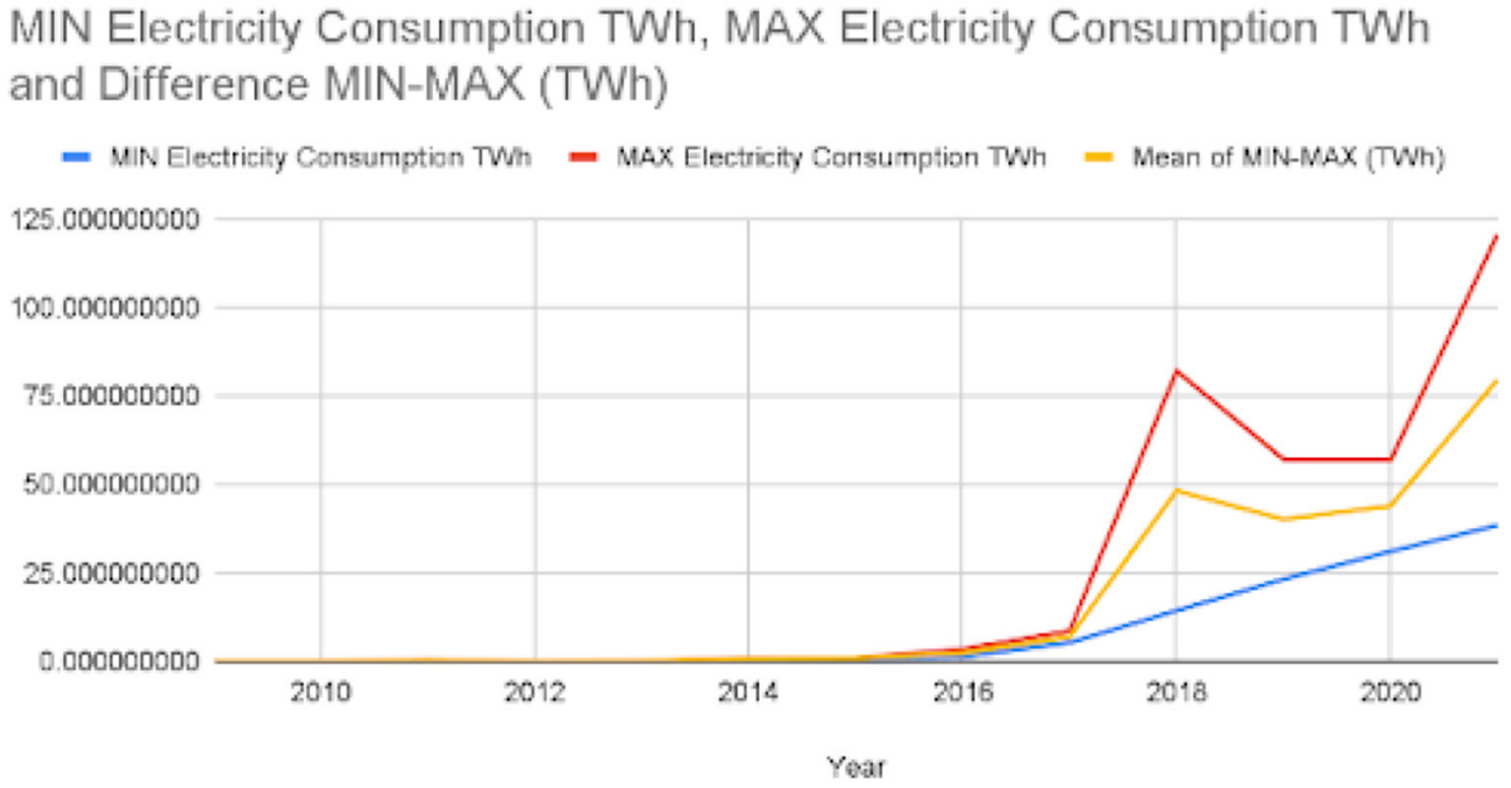
MIN, MAX, and MEAN electricity consumption of bitcoin mining in Tera-watt-hour from year 2011 to 2021.

**Figure 6:**
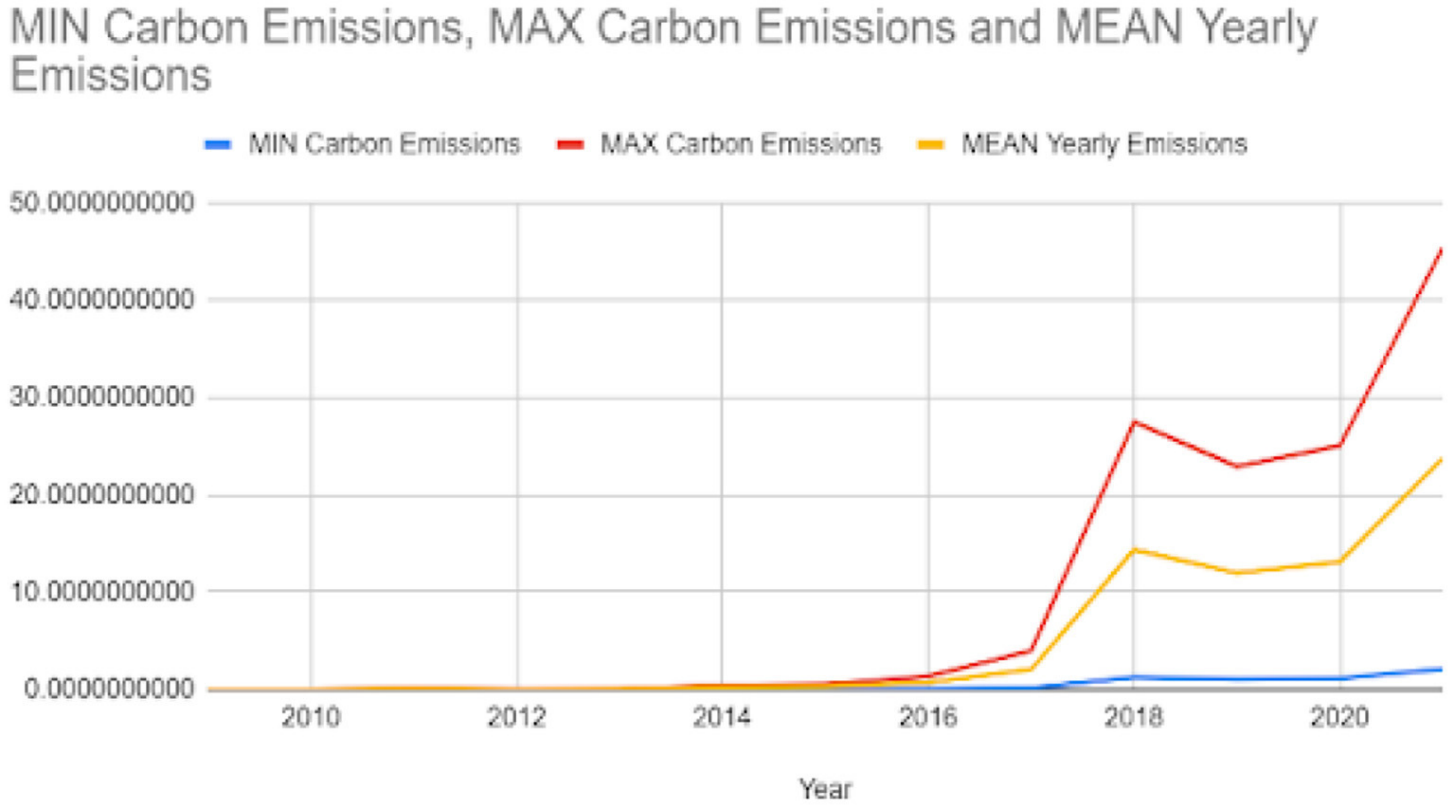
MIN, MAX, and MEAN carbon emission of bitcoin mining in Tera-watt-hour from year 2011 to 2021.

**Figure 7:**
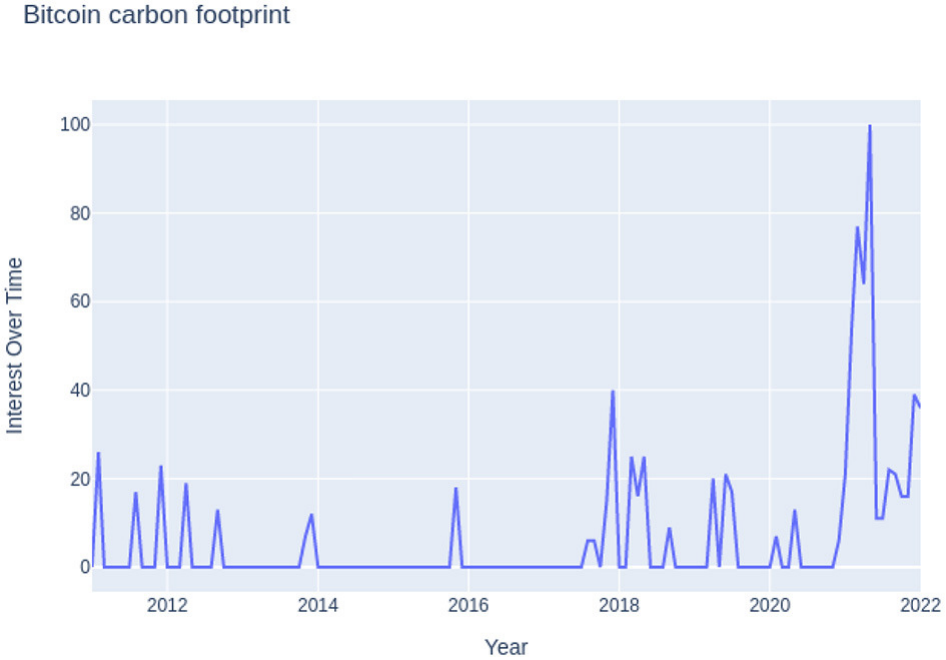
Google searches for “Bitcoin Carbon Footprints” from 2011 to 2021 (Google Trends, 2022b).

For the year 2021, it was computed that bitcoin mining released between 2.12 and 45.37 million metric tons of carbon. The total carbon emission was also estimated in the range of 5.95 to 127.372; again, the exact number cannot be estimated due to a lack of miners' data. A graph was plotted for carbon emissions and electricity consumption for bitcoin mining from 2011 to 2021 and is shown in Figures 5, 6. These figures show a decline in electricity consumption and carbon emissions. This can be explained by the fact that mining machines are getting better and use less electricity. This decline, however, did not last long, as cryptocurrency became more popular beginning in 2020. Therefore, the public started trading and mining Bitcoin and other cryptocurrencies. Meanwhile, 2018 acted as a wake-up call for miners as it raised concerns about the environment in the miners' community (Google Trends, 2022a,b). Figure 7 shows that the number of Google searches for “Bitcoin Carbon Footprint” is going up, which is in line with what Table 4 shows.

Table 6 presents the percentage change in electricity consumption over the years 2011–21. Table 6 and Figure 4 depict the growth in popularity and technology over the lifecycle of bitcoin from 2011–21. The first lucrative year for Bitcoin was 2012, which also saw a drop in electricity use of roughly 2021.78 percent. This was because miners started to treat Bitcoin mining as a job rather than just another pastime.

**Table 6:** Percentage Increase in annual consumption of electricity for bitcoin mining.

**Year**	**Percentage change in electricity consumption**
2011	99.83%
2012	−2021.78%
2013	87.92%
2014	90.56%
2015	19.03%
2016	59.41%
2017	65.72%
2018	85.50%
2019	−20.03%
2020	8.62%
2021	44.75%

During this time, Field Programmable Gate Arrays (FPGAs) became popular for the sole purpose of Bitcoin mining. In the year 2019, the amount of electricity used dropped by 20%. This could be because mining machines are getting more efficient or using less energy. In 2021, there is a final increase of almost 45%. This could be because of the COVID-19 outbreak when most investors started to put their money into cryptocurrencies and Web3.0 as the industry started to grow (Béjaoui et al., 2021).

## 5. Discussion

There has been a lot of discussion about how much power bitcoin mining uses, and different ways have been suggested in the literature to estimate it. However, these approaches have a few limitations, as they only provide an estimated consumption value for electricity. Zumo methodology focuses on the average amount of electricity required to mine one coin (Gallersdörfer et al., 2021). It calculates energy consumption using the value of the miner's revenues and the average unit price of electricity. The revenue earned by the miners to maintain the blockchain is calculated by adding the block reward of the cryptocurrency and the associated transaction fees. The power consumption is calculated by dividing sixty percent of the miner's revenue by the average unit price of electricity. Patch methodology calculates the daily electricity consumption of cryptocurrency mining using commercially available mining equipment and their efficiencies (Li et al., 2019). The amount of power used is found by multiplying the efficiency of the hardware with the network hash rate of the currency. Using the Zumo methodology, the power consumption was estimated to be 200 TWh for the year 2021, while it was estimated as 60 TWh using the Patch methodology. However, these findings do not appear to be comparable to the existing literature. Furthermore, Zumo methodology employs assumptions (assuming that 60% of revenue is spent on power) to compute the results, raising concerns about the accuracy of the data. It is important to find a better method for calculating a more realistic value of power usage that doesn't rely on assumptions. The patch methodology utilizes hardware efficiency and the geographical location of miners to calculate a single figure of electricity consumption and carbon footprint, which may not be accurate. It is infeasible to measure exactly how well mining hardware works because miners are spread out all over the world and use different sources of electricity.

Instead of using the miners' location and the source of electricity production, this work has proposed best and worst-case scenarios for the efficiency of the hardware as well as sources of power generation. The narrow range is obtained for electricity consumption and carbon emissions, which are in line with the results reported in the literature.

## 6. Conclusion

A smart city is one of the greatest demands of a modern nation. It can adapt to rapidly changing technology for its sustenance. It uses modern technologies to raise the general standard of living for the masses. Cryptocurrency is one such pioneering currency of the twentifirst century that uses blockchain technology. The initial purpose of the technology was to simplify the electronic movement of money. But in the last few years, the blockchain has grown into a reliable technology that can be used in many different ways. Bitcoin, being one of its kind, empowers journalists, activists, and many other professionals all over the world. These people are always under the scrutiny of an organization or two, and blockchain helps them receive funds without the intervention of any overseeing authority. Also, because the transactions on the blockchain network can't be tampered with, it protects smart cities from cyberattacks. The primary concern is how getting rid of all fiat currency and all of its worldwide transactions will affect the environment. The proposed work was used to look into how Bitcoin mining affects the environment. Since Bitcoin has become more efficient over time, electricity use and carbon emissions have gone down. Mining machines, which are the main source of pollution, have gotten better over time, which has made them use less electricity. Using the proposed method, it was estimated that Bitcoin used between 38.495 and 120.728 terawatt hours of electricity in 2021, while it emitted between 2.12 and 45.379 million metric tons of carbon.

Many individual miners and mining pools are also shifting to environment-friendly and cost-effective means of energy like solar panels. However, a fair number of mining pools are already concentrating around energy-intensive areas like aluminum mining sites, which use hydroelectricity for energy generation. Proof-of-stake is another alternative to the current system. People think that the proof-of-work consensus mechanism used to verify transactions is the main reason why Bitcoin uses so much energy. But compared to the current method, it is still fairly new, and proof-of-stake is not as good or reliable as proof-of-work because it depends too much on the goodwill of the stakeholders (Poelstra, 2015). There is still much work to be done before Bitcoin can completely replace the current financial system in smart cities.

## Data availability statement

The original contributions presented in the study are included in the article/supplementary material, further inquiries can be directed to the corresponding author.

## Author contributions

All authors listed have made equal contribution to the work and approve it for publication.
